# The Burden of Typhoid Fever in South Africa: The Potential Impact of Selected Interventions

**DOI:** 10.4269/ajtmh.18-0182

**Published:** 2018-07-25

**Authors:** Karen H. Keddy, Anthony M. Smith, Arvinda Sooka, Nomsa P. Tau, Hlengiwe M. P. Ngomane, Amruta Radhakrishnan, Daina Als, Frew G. Benson

**Affiliations:** 1 Centre for Enteric Diseases, National Institute for Communicable Diseases, Johannesburg, South Africa;; 2 Faculty of Health Sciences, University of the Witwatersrand, Johannesburg, South Africa;; 3 Centre for Global Child Health, The Hospital for Sick Children, Toronto, Canada;; 4 Gauteng Provincial Health Department, Johannesburg, South Africa

## Abstract

Typhoid fever is notifiable in South Africa but clinical notification is notoriously poor. South Africa has an estimated annual incidence rate of 0.1 cases per 100,000 population of culture-confirmed typhoid fever, decreased from 17 cases per 100,000 population in the 1980s. This work was undertaken to identify the reasons for this decrease and identify potential weaknesses that may result in an increase of observed cases. Culture-confirmed cases, with additional demographic and clinical data have been collected from selected sentinel sites since 2003. Data on contextual factors (gross domestic product [GDP], sanitation, female education, and childhood diarrhea mortality) were collected. National incidence rates of culture-confirmed typhoid fever have remained constant for the past 13 years, with the exception of an outbreak in 2005: incidence was 0.4 per 100,000 population. Paratyphoid fever remains a rare disease. Antimicrobial susceptibility data suggest resistance to ciprofloxacin and azithromycin is emerging. The South African population increased from 27.5 million in 1980 to 55.0 million in 2015: urbanization increased from 50% to 65%, GDP increased from United States Dollar (USD) $2,910 to USD $6,167, access to sanitation improved from 64.4% to 70.0% in the urban population and 26.4% to 60.5% in rural areas. Female literacy levels improved from 74.8% to 92.6% over the period. Improved socioeconomic circumstances in South Africa have been temporally associated with decreasing incidence rates of typhoid fever over a 35-year period. Ongoing challenges remain including potential for large outbreaks, a large immigrant population, and emerging antimicrobial resistance. Continued active surveillance is mandatory.

## SIGNIFICANCE OF THIS STUDY

Typhoid fever is notifiable disease in South Africa, an emerging economy, but clinical notification is notoriously poor. The estimated annual incidence rate of typhoid fever is 0.1 cases per 100,000 population, which has decreased since the 20th century. Currently, typhoid fever cases are primarily notified through a laboratory-based notification system to the national reference laboratory which confirms the identity of the bacteria causing typhoid fever and paratyphoid fever. National incidence rates of culture-confirmed typhoid fever have remained constant around 0.1 per 100,000 population for the past 13 years but increased to 0.4 per 100,000 population in an outbreak in 2005; Paratyphoid fever remains a rare disease. Antimicrobial susceptibility data suggest that resistance to ciprofloxacin and azithromycin is emerging. Improvements in the gross domestic product (GDP), sanitation, and female literacy are believed to have contributed to decreasing incidences but emerging multidrug resistance and the potential for outbreak have highlighted the importance of ongoing surveillance. This study tracks those interventions that may have affected the incidence rates, examines why these are not decreasing further and suggests further studies and interventions that may combat typhoid fever in South Africa, including targeted vaccination programs, cross-border cooperation, and ongoing social improvements.

## INTRODUCTION

Recent evidence has shown that South Africa has low endemicity for typhoid fever, with an estimated annual incidence rate of culture-confirmed disease of 0.1 cases per 100,000 population,^1^ nonetheless, outbreaks occur in vulnerable populations, such as those living in informal settlements or in crowded conditions.^2,3^ Previous studies have highlighted that 50 years ago, there was a very different pattern of disease, and in parts of the country, typhoid fever was highly endemic, with an estimated blood culture positivity rate of 500 per 100,000.^4^ Nonetheless typhoid fever and outbreaks in particular remain a problem on the Southern African subcontinent, with outbreaks having been reported from Zimbabwe, Zambia, and Malawi.^5–8^

The current national surveillance system for typhoid and paratyphoid fever in South Africa relies on two methods. Firstly, enteric fever is a notifiable disease in South Africa and every case is required by law to be reported to the National Department of Health (NDoH). However, in 2007, Weber showed that although legally required, only 37% of general practitioners in the predominantly urbanized population of Gauteng Province, South Africa, comply.^9^ Secondly, the notification system is supported by a laboratory-based surveillance system, which has been supplemented in part by enhanced surveillance for enteric fever at selected sentinel sites.^1^ In addition to confirming each culture-positive case of typhoid fever, this latter system collects information on clinical presentation, antimicrobial susceptibility patterns, and molecular epidemiology of *Salmonella enterica* serotype Typhi (*Salmonella* Typhi)^10^ and *Salmonella* Paratyphi A, *Salmonella* Paratyphi B, and *Salmonella* Paratyphi C (*Salmonella* Paratyphi) isolates.^11^

### Historical patterns of typhoidal *Salmonella*.

Review of the national notifiable disease data in South Africa before 1990 suggest that typhoid fever rates were much higher in the country, with approximately 4,000 cases being notified annually, based on clinical, serological or microbiologically confirmed diagnoses,^12^ although these rates decreased after 1990.^13^ Some of this decrease could be ascribed to underreporting of enteric fever cases.^9^ Despite a comparatively robust surveillance system, complications, such as typhoid ileal perforations, have not been well described in South Africa. Previous studies have reported disproportionately high rates of ileal perforations among women, which is contrary to what has been noted in other African countries, where this is rarely described.^14–16^ A recent publication has suggested that excessive mortality may occur in human immunodeficiency virus-infected (HIV) patients in South Africa with typhoid fever but data could not confirm that HIV infection was a risk factor for typhoid fever.^1^

Conversely, paratyphoid fever appears to be a rare disease in this country.^11,17^ In a limited clinical series, collected through the GERMS-enhanced surveillance network (previously an acronym for Group for Enteric Respiratory and Meningeal disease in South Africa [GERMS-SA]), no cases of paratyphoid fever died,^11^ although mortality due to typhoid fever remains high.^1^ Between 1945 and 1977, the case fatality rate (CFR) due to typhoid fever reportedly decreased from 15.5% to 1.1% but recent laboratory-based surveillance studies between 2003 and 2013 suggested that this mortality may be around 6.8%.^1^

### Typhoid control measures.

Improving social and political circumstances in South Africa have had a dramatic effect on the health of the population, although this has been tempered by an extremely high HIV prevalence, estimated at 15% of the adult population in 2013.^18^ Nonetheless, as a notifiable condition, typhoid fever outbreaks receive significant attention and active case finding and contact tracing is the norm under such conditions. However, despite active campaigns for safe water, approximately 17% of the South African population do not have access to safe water as defined by the national Reconstruction and Development Program (RDP) standards, specifically 6 kiloliters (kL) per household per month at a radial distance of 200 m.^19^ Access to sanitation, (defined as at least a ventilation-improved pit latrine^19^) is lagging as well and, according to RDP standards is available to 70% of the country’s population; however, only 38% of the population of Limpopo Province, bordering on Zimbabwe and Mozambique, have adequate access to sanitation,^19^ highlighting regional discrepancies (Supplemental Table 1). Regulations for the monitoring of food handlers for carriage of typhoid fever are in place, including the inspection of premises to ensure provision of handwashing facilities, provision of protective clothing, and testing of suspected carriers in the event of an outbreak.^20,21^ Currently, however, vaccination against typhoid fever is not recommended for routine use among food handlers. Most of the population has had at least a primary school education (www.statssa.gov.za) but the social situation is complicated by a large immigrant population from neighboring countries, from which typhoid fever outbreaks have recently been reported.^22,23^

Guidelines for the management of typhoid fever have been developed by the National Institute for Communicable Diseases and are regularly updated, depending on the occurrence of outbreaks and changing antimicrobial resistance patterns.^24^ These guidelines include the use of blood culture rather than rapid tests for diagnosis, due to loss of important information on antimicrobial susceptibility, the use of ciprofloxacin in adults and azithromycin in children for susceptible isolates and reminder for notification of typhoid fever cases, for the purposes of contact tracing.

This work was undertaken to better understand what factors influenced the marked decrease in the number of typhoid fever cases in South Africa over the past few decades and identify potential problems and weaknesses in the national systems that may result in increases in endemic and epidemic cases in the country.

## METHODS

### Data on typhoidal *Salmonella*.

Historical data on typhoid fever incidence rates, based on clinical notifications, were derived from Küstner and the NDoH epidemiological comments.^12,13^ *Salmonella* Typhi and *Salmonella* Paratyphi (A, B, and C) isolates from diagnostic laboratories identified in South Africa from 2003 to 2015 were submitted to the national reference laboratory as part of a national surveillance program (GERMS-SA) for isolate confirmation of notifiable medical conditions. Systematic surveillance in 2014 and 2015 was stopped because of financial and managerial constraints. Surveillance included both invasive and noninvasive (fecal specimens) isolates. *Salmonella* Typhi and *Salmonella* Paratyphi isolates were confirmed biochemically and serotyped according to standard operating procedures, based on the White–Kauffmann–Le Minor typing scheme. Antimicrobial susceptibility testing (minimum inhibitory concentrations [MIC]) against ampicillin, chloramphenicol, ciprofloxacin, and ceftriaxone was carried out between 2003 and 2013; testing for susceptibility to azithromycin, in conjunction with ciprofloxacin, was introduced systematically from 2012 on all *Salmonella* Typhi isolates.^25^ Additional clinical data were sourced from selected sentinel sites as part of a national surveillance initiative, GERMS-SA, which ran between 2003 and 2013.^1^ Molecular epidemiological characterization using pulsed-field gel electrophoresis (PFGE) was undertaken on all isolates using PulseNet protocols.^26^ Data were supplemented by audits of the Central Data Warehouse of the National Health Laboratory Service, a centralized database that stores results of all pathology tests carried out at state health facilities in South Africa. Molecular subtyping using PFGE analysis was routinely introduced in 2010 for all cases confirmed microbiologically at the national reference center, including retrospective PFGE analysis on archived isolates received from 2003 to 2009, and is performed on all *Salmonella* Typhi and *Salmonella* Paratyphi (including *Salmonella* Paratyphi A, *Salmonella* Paratyphi B, and *Salmonella* Paratyphi C) isolates.

### Data on contextual factors that may have intervened in typhoid fever incidence rates.

Data on the numbers of public sector medical practitioners per 100,000 uninsured population per province were accessed from the Heath Systems Trust.^27^ Data on access to safe water and sanitation were obtained through accessing reports published by the World Health Organization/United Nations Children’s Fund (WHO/UNICEF) Joint Monitoring Program (JMP) for Water Supply and Sanitation, World Bank, Development Research Group and a report on national water projects undertaken by the University of Pretoria.^28,29^ Data were based on primary household surveys obtained from government statistical agencies and World Bank country departments, based on United Nations Educational, Scientific, and Cultural Organization Institute for Statistics, WHO/UNICEF JMP for Water Supply and Sanitation.^28,30^ Data before 1990 was calculated using the Forecast function in Microsoft Excel. Data on education were accessed from the Demographic Health Survey (2003). A large typhoid fever vaccine trial in South Africa in the mid-1980s was reviewed: 23,000 children aged between 5 and 16 years were enrolled^4^ and the trial was conducted in an area that served 3.7 million people, of whom one million fell within the age group vaccinated.^18^ Information was gathered on a large project that was simultaneously undertaken to provide safe water in the area.^28^

### Statistical methods.

National incidence rates of culture-confirmed typhoid fever from 2003 to 2015 were calculated based on national population estimates provided by the Department of Statistics.^30^ The number of cases of *Salmonella* Paratyphi was considered to be too low to derive meaningful incidence estimates.

## RESULTS

### Trends in typhoidal *Salmonella* (2003–2015).

**Figure 1.**
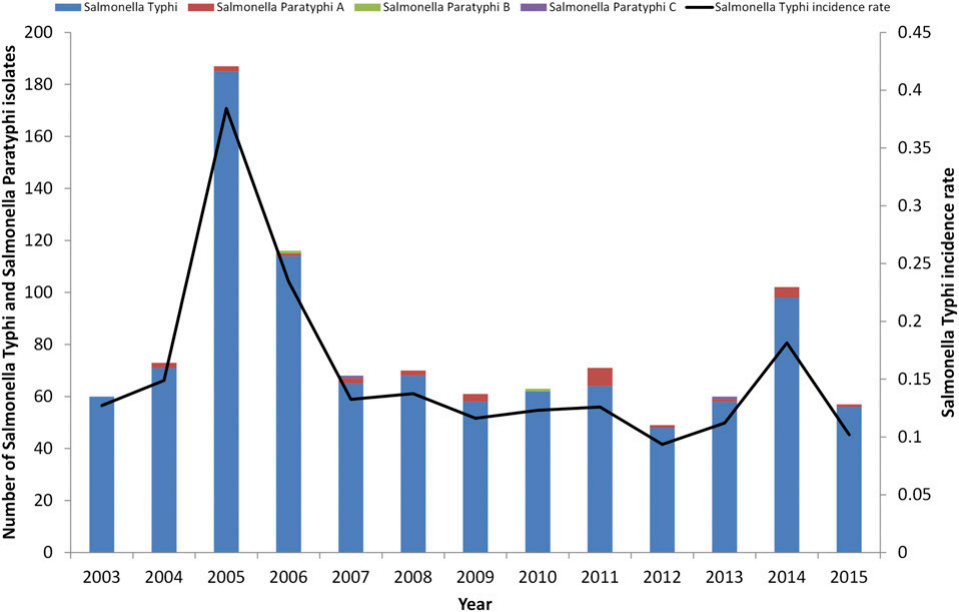
Trends in invasive culture-confirmed typhoidal *Salmonella* over time, 2003–2015: *Salmonella enterica* serotype Typhi (*Salmonella* Typhi [*N* = 1,007], *Salmonella* Paratyphi A [*N* = 26], *Salmonella* Paratyphi B [*N* = 2], and *Salmonella* Paratyphi C [*N* = 2]). Increased cases numbers in 2005 are due to an outbreak that occurred in that year.^1^

Annual culture-confirmed isolation rates of invasive *Salmonella* Typhi and Paratyphi are illustrated in Figure 1. Over the study period, 1,007 *Salmonella* Typhi isolates, 26 *Salmonella* Paratyphi A isolates, and two isolates, respectively, of *Salmonella* Paratyphi B, and *Salmonella* Paratyphi C were identified. (Supplemental Figure 1 shows decreasing typhoid fever incidence rates, based on notifications, between 1985 and 2005, while tracking selected interventions. Annual incidence rates of typhoid fever subsequent to 2003 remained low, from 0.1 to 0.2 cases per 100,000 population, with the exception of 2005, when an increase to 0.4 per 100,000 was observed in connection with a documented outbreak (Figure 1) (see Keddy et al. for a detailed analysis and map).^1^

Mortality data for culture-confirmed cases were available from enhanced surveillance sites from 2003 to 2013 only: 16/237 (6.8%) patients died.^1^ Major risk factors for mortality were coinfection with HIV, disease severity and delayed presentation to hospital.^1^ Data on perforations were not recorded.

### Patterns of antimicrobial resistance.

**Figure 2.**
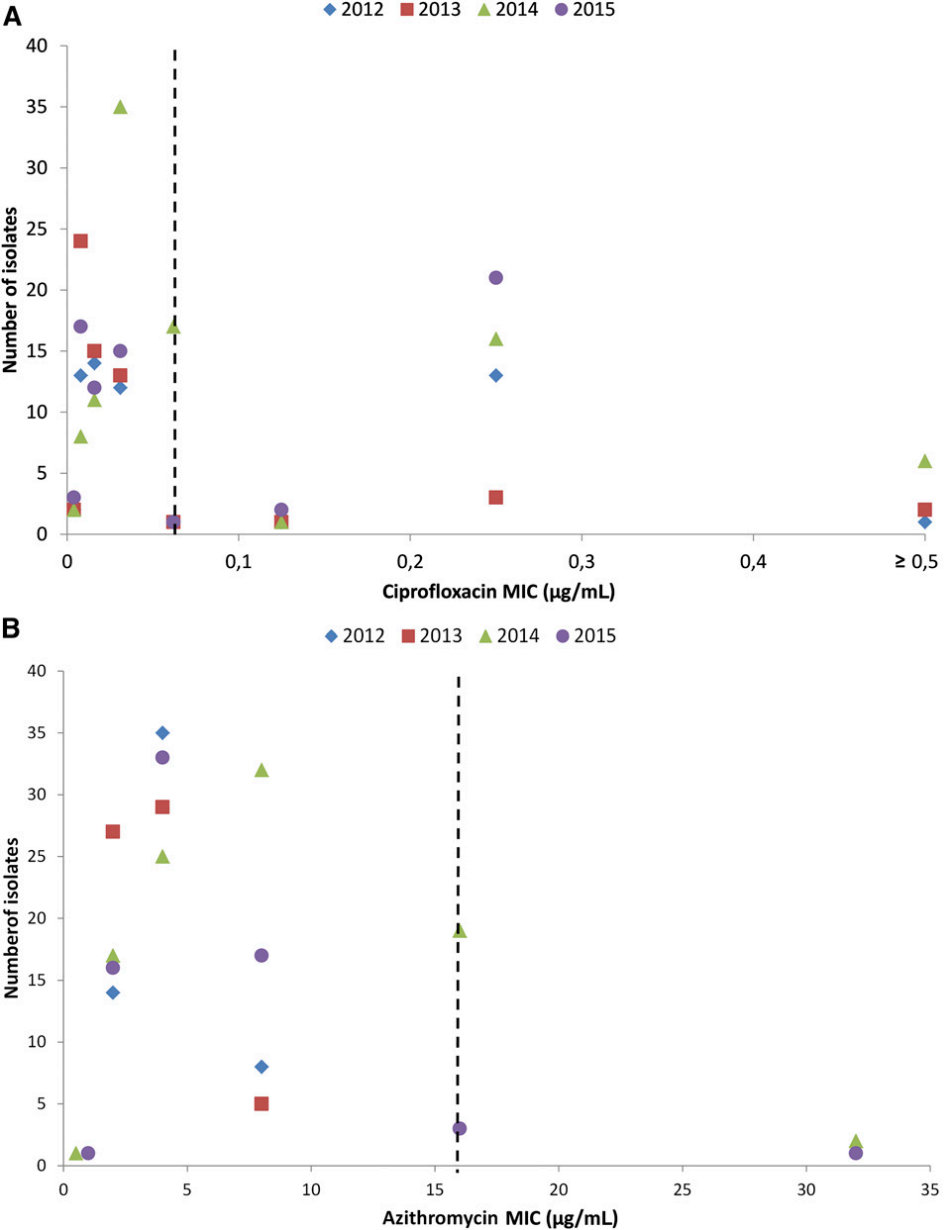
(**A**) Minimum inhibitory concentrations (MICs) of *Salmonella* Typhi isolates to ciprofloxacin. Scattergram showing increasing MICs (right shift) of *Salmonella enterica* serotype Typhi (*Salmonella* Typhi) (*N* = 285) to ciprofloxacin, 2012–2015. The dotted line represents the ciprofloxacin breakpoint (0.06 μg/mL). (**B**) Minimum inhibitory concentrations of *Salmonella* Typhi isolates to azithromycin Scattergram showing increasing MICs (right shift) of *S. enterica* serotype Typhi (*Salmonella* Typhi) (*N* = 285) to azithromycin, 2012–2015. The dotted line represents the azithromycin breakpoint (16 μg/mL).

Susceptibility data for *Salmonella* Typhi between 2003 and 2013 and *Salmonella* Paratyphi between 2003 and 2014 have previously been published.^1,11^ Figure 2A and B are scattergrams representing annual trends in ciprofloxacin and azithromycin resistance, respectively, for all culture-confirmed isolates of *Salmonella* Typhi, from 2012, when systematic testing for azithromycin was initiated, to 2015. Data for both ciprofloxacin and azithromycin reveal increases in the MICs to both antimicrobials over the period from 2012 to 2015. Because low numbers of *Salmonella* Paratyphi were isolated, formal analysis of resistance trends could not be undertaken.

### Molecular epidemiology.

For the years 2003–2015, PFGE analysis was performed on 917 *Salmonella* Typhi isolates to establish a database of diverse PFGE patterns, which has proved invaluable in relating isolates and monitoring for emergence of new strains and clusters. Pulsed-field gel electrophoresis analysis has confirmed importation of typhoid cases from neighboring countries and overseas countries, as well as assisted in local outbreak investigations (Supplemental Figure 2).^31,32^ Representative PFGE patterns from major South African clusters have been submitted to the Global PulseNet *Salmonella* Typhi Database hosted by PulseNet USA. For *Salmonella* Paratyphi, PFGE patterns for all isolates to date (*N* = 42) have been captured.^11^ Going forward, PFGE analysis will be augmented by whole-genome sequencing (WGS) analysis of selected isolates, as required for investigation of clusters and outbreaks.

### Trends in contextual factors and qualitative data.

The South African population is currently estimated to be around 55 million people, increasing from 27.5 million in 1980.^30,33^ Over this period there has been significant migration from rural to urban areas. In 1980, it was estimated that approximately 50% of the population lived in urban areas compared with 65% in 2015.^30,33^ The GDP increased from $2,920 USD per capita to $6,167 USD per capita in 2013.^33^ The projected health-care expenditure in the government sector in 2013/2014 was South African Rand (ZAR) 141.7 billion (48.2% of total health-care expenditure), approximately USD $11.8 billion, including laboratory tests e.g., blood cultures. In contrast, projected expenditure was roughly equivalent at ZAR 146.6 billion (49.9% of total health-care expenditure), approximately USD $12.2 billion in the private sector, covering approximately 17% of the population in 2013/2014, most of whom were on some form of medical insurance,^34^ which would include the costs of laboratory-based tests. The number of culture-confirmed typhoid fever cases per province was compared with the number of public sector medical personnel per 100,000 uninsured population, in South Africa from 2003 to 2015 for provinces with higher incidence rates (Supplemental Figure 3) and provincial expenditure per capita on laboratory diagnostics (Supplemental Table 1).

Between 1980 and 2015, there were marked improvements in accessibility to sanitation in South Africa. In urban areas, accessibility rose from 61.4% in 1980 to 70.0% in 2015, compared with rural areas where this rose from 26.4% to 60.5% over the same period.

**Figure 3.**
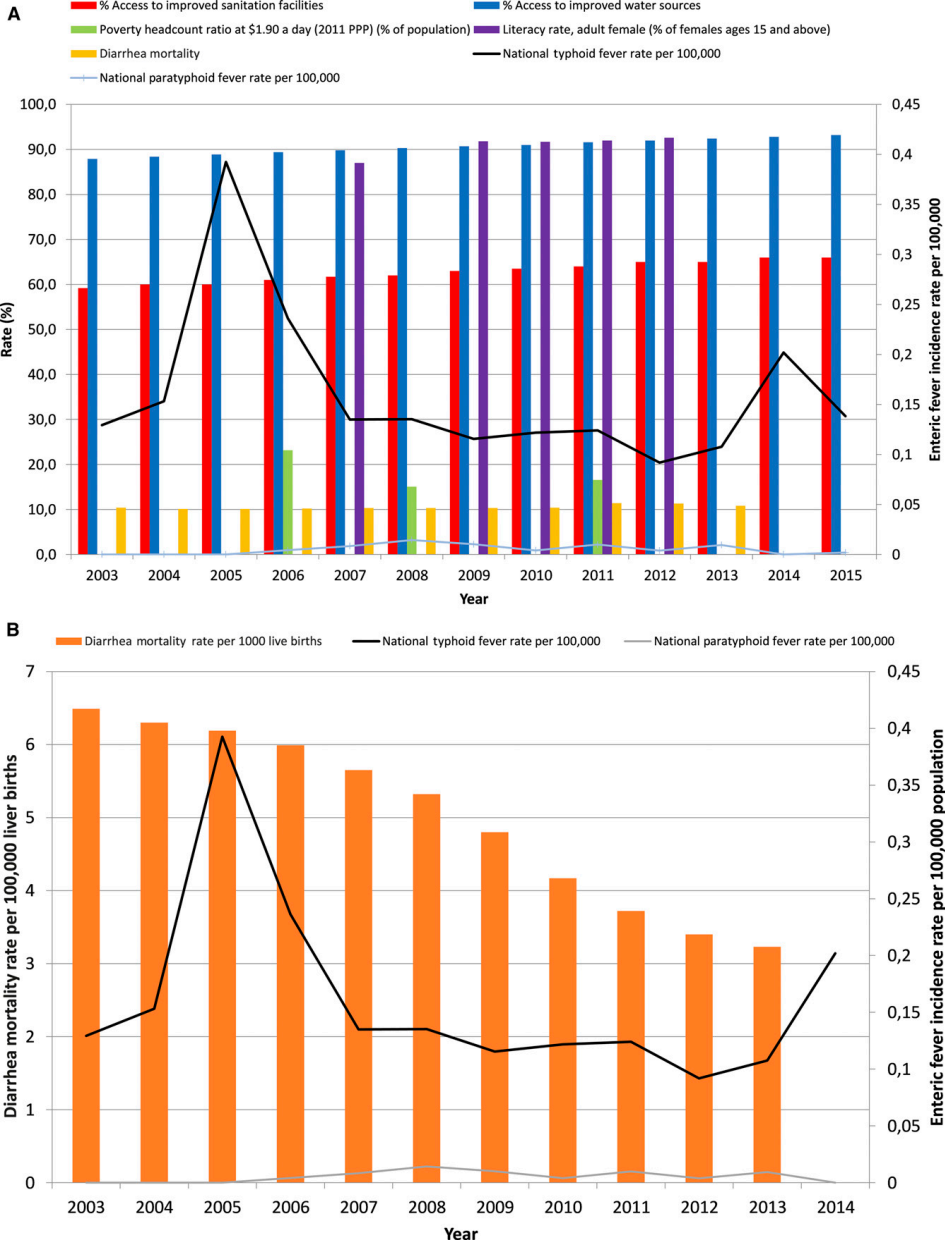
(**A**) Contextual factor trends, including under-five mortality, poverty head count, access to improved water sources and female literacy in South Africa. (**B**) Changing diarrhea mortality per 1,000 live births, national typhoid fever rate and paratyphoid fever rate in South Africa. Trends in contextual factors over time, reflecting changing socioeconomic circumstances for the South Africa population between 2003 and 2015, compared with annual incidence rate of typhoid fever per 100,000 population.

Between 1993 and 2011 the number of South African residents living below the breadline (< USD $1.90 per day) increased from 31.9% to 35.2% in 2000 but then dropped dramatically to 15.1% in 2008, increasing slightly again in 2011, to 16.6%. There was also an increase in adult female literacy over the time period, rising steadily from 74.8% in 1980 to 92.6% in 2012. Nonetheless, between 2003 and 2015, there was little change in disease burden due to typhoid fever in South Africa, despite ongoing trends in improvements in socioeconomic circumstances (Figure 3A and B). Similarly, between 2003 and 2013, childhood mortality due to diarrhea remained constant around 10%.

Typhoid fever vaccination is not part of the current immunization strategies in South Africa,^35^ although the country was previously the site of a large trial to assess the protective efficacy of the Vi (capsular polysaccharide) vaccine in the 1980s.^4^ In the area in which the vaccination program was undertaken (Limpopo Province), 0.6% of children aged between 5 and 16 years were vaccinated for typhoid fever, a further 0.6% received Meningococcal A+C vaccine and the remainder of those enrolled (∼11,000) received neither.

## DISCUSSION

### Overall pattern of typhoidal *Salmonella*.

We have observed a dramatic decrease in the number of typhoid fever cases in South Africa being reported between the incidence rates from notified cases to the NDoH in the 1980s^13^ and those confirmed by laboratory-based surveillance for typhoid fever from 2003 onwards. A wide variation between the incidence rates in different provinces was noted between 2003 and 2015. Data from 2014 to 2015 may be further underrepresenting the true case numbers, as systematic laboratory-based surveillance was stopped in these years. This is likely a reflection of health-care practices, rather than a true reflection of endemic typhoid fever, as it correlates with a higher number of medical personnel available in those provinces from which culture-confirmed typhoid fever is more frequently reported. In addition, as these provinces (Gauteng Province and Western Cape) are recognized as the most sophisticated in South Africa, health-care seeking behavior potentially results in more patients presenting to hospitals here for treatment, although typhoid fever may be acquired elsewhere. Last, although there are guidelines for the diagnosis and management of typhoid fever that are regularly updated, these lack a specific clinical case definition for typhoid fever and instead define cases as “case under investigation” or (microbiologically) “confirmed case.”^24^ Thus, clinically, any patient with documented fever ≥ 38°C, with a recent travel history to an area where there is a known typhoid fever outbreak, or in addition presents with the classic accompanying symptoms (rose spots, relative bradycardia, hepatomegaly, or splenomegaly etc.) would be regarded as a “case under investigation”: in the absence of blood cultures, such patients may receive empiric antimicrobial therapy but not be reported. As the highest rates of private health-care insurance and the greatest numbers of medical schools are in the Western Cape and Gauteng provinces,^34^ typhoid fever case numbers may be skewed by diagnostic practices that are greater in those provinces where health-care practice is perceived to be better and blood cultures are more likely to be carried out. Mortality data may equally be skewed as these reflect data collected from a subset of hospitals only, which typically have academic links.^1^

### Factors that may have contributed to changes in typhoidal *Salmonella*.

Although most of the data analyzed here focus on the time period from 2003 to 2015, the rapid socioeconomic improvements in the latter two decades of the 20th century in South Africa potentially had a long term effect on typhoid fever incidence. These changes, thus, may have occurred in conjunction with the improvement in accessibility to safe water in South Africa from 1980 to 2002 (Supplemental Figure 1) and the introduction of sanitation, particularly in rural areas, from where the highest rates of typhoid fever were previously reported.^12^ Improved socioeconomic circumstances, as suggested by both the improvement in adult female literacy and the decrease in the numbers of people living below the breadline, may also have been a contributing feature. Data on contextual factors, being derived from official reports, were deemed not sufficiently robust for statistical analyses.

Improvements in socioeconomic circumstances and general living conditions have likely contributed considerably to decreasing typhoid fever rates. A recent increase in the numbers of cases in the country could be attributed to large numbers of economic migrants entering the country illegally, rather than due to local transmission of disease.^36^ Nonetheless, health disparities may be masking true incidence rates, as manifested by the severe lack of medical personnel (< 1 per 1,000 population) in South Africa in general and certain provinces, such as Limpopo and North West, in particular. Recent WHO reports recommend 4.1 skilled medical professionals per 1,000 population and a shortfall may result in underutilization of diagnostic services.^37^ Although South Africa has the highest health worker density in sub-Saharan Africa (5.9 per 1,000 population),^37^ there is nonetheless a shortfall in certain provinces. In a country with a sophisticated road infrastructure, this has affected health-care–seeking behavior and patients tend to travel to better resourced provinces for their medical care.^1^ We used medical practitioners in the public sector as a proxy, as in South Africa, this group is the most likely to be directly involved in laboratory-based diagnosis of enteric fever.

Additional decreases in incidence rates may have occurred due to modification in the recommended antimicrobial management policies, with the introduction of ciprofloxacin for typhoid fever treatment in the 1990s and the addition of azithromycin to the treatment protocol after 2008. These antimicrobials have the added advantage that they can be used over a shorter treatment period.^38,39^ Both ciprofloxacin and azithromycin appear to be associated with lower carrier rates for *Salmonella* Typhi and usage may have decreased the infective pool. A point of concern to note is that, every year from 2012 to 2015, since testing for azithromycin was introduced in the reference laboratory; greater numbers of *Salmonella* Typhi isolates from South African patients are approaching the respective breakpoints for resistance to both ciprofloxacin and azithromycin, or testing as intermediately or fully resistant. Low case numbers, however ,prevented a comprehensive analysis for the comparable challenge described in Nepal.^40^ If this trend continues, in a few years, most infections due to *Salmonella* Typhi in South Africa may not respond to currently recommended therapy. Previous work has shown that antimicrobial treatment regimens for typhoid fever in South Africa are robust, and mortality is associated with treatment delays due to late hospital presentation, severe disease, or HIV infection.^1^ This CFR is six times that reported by Küstner based on national surveillance data in 1977^18^ and may be skewed by both the additional risk posed by HIV coinfection^1^ and the inclusion of primarily tertiary care facilities in the sentinel surveillance system.

It is difficult to determine whether the typhoid vaccine trial in the 1980s contributed in any way to the decrease in the burden of disease due to typhoid fever^4^; although the trial was undertaken in an area that purportedly had the highest reported rates of typhoid fever in South Africa at the time, that is, six times the national average.^12^ There were also major programs to provide safe water in the area, which could have in addition impacted disease rates.^4,29^ Going forward, vaccine programs may encompass targeted vaccination of individuals in areas at risk for typhoid fever outbreaks, particularly with the problem of emerging multidrug resistance.

### Limitations in data and analyses.

Major data gaps in understanding typhoid fever remain: mortality data are not systematically collected and the true burden of complications, including both intestinal perforation and neurological sequelae,^7^ are unknown. Resource limitations have resulted in cutting back on the number of confirmatory tests at the national reference laboratory, so a sequential analysis of changes in antimicrobial resistance from 2003 to 2015 could not be undertaken. Complete data on socioeconomic circumstances were also not available for the time period 2003–2015 and surveillance data for typhoid fever for the decades before this period were sparse, so a complete analysis could not be undertaken for either period. Published data from the NDoH on the number of typhoid fever cases may be lacking as not all cases are notified; thus, we do not know how many typhoid fever cases may be diagnosed using serological or purely clinical methods and these data have not been published since 2008. In addition, typhoid fever diagnostics using serology has performed poorly in South Africa in the past.^41^ We attempted to use the number of medical practitioners as a proxy to better define those provinces where diagnostics may potentially be weak but recognize that this linkage may be ecological, rather than cause and effect.

It appears that paratyphoid fever is rarely diagnosed in South Africa, and even through the national laboratory-based surveillance system, we did not see the numbers reported from earlier studies on paratyphoid fever, undertaken in the 1990s.^17^ With the paucity of data that we currently have available, we thus could not adequately postulate whether socioeconomic or microbiological factors have equally impacted disease rates of paratyphoid fever.

### Outstanding questions.

From a public health perspective, enhanced patient follow-up and contact tracing should reveal where *Salmonella* Typhi infection was acquired, identifying whether disease is locally acquired or imported from neighboring provinces or countries. Major advances have been made in this area over the past 2 years,^36^ but this increased vigilance needs to be sustained to truly understand the epidemiology of typhoid fever in South Africa. The impact of shortages of health personnel among the uninsured population and in specific provinces needs to be better elucidated to derive better estimates of the true burden of typhoid fever. Better data on the burden of antimicrobial resistance and the presence of *Salmonella* Typhi H58 need to be assessed. *Salmonella* Typhi H58 is a highly clonal multidrug-resistant haplotype of *Salmonella* Typhi that is being reported with increasing frequency from many countries in Africa and Asia.^42,43^ Continual antimicrobial resistance monitoring has become an essential adjunct to assessing how best to control typhoid fever.^44^ Molecular analyses, including PFGE and more recently WGS have confirmed that South Africa remains highly vulnerable to the introduction of multidrug-resistant typhoid fever and potential outbreaks that may result from this,^31,32^ particularly as ongoing outbreaks of fluoroquinolone-resistant typhoid fever are reported from neighboring countries.^45^

### Way forward.

Typhoid fever control in South Africa will rest primarily on active monitoring of cases, including follow-up of patients to exclude carriage and contact tracing. Given the emergence of antimicrobial resistance, there may be a role for limited vaccination campaigns in areas where typhoid fever may be perceived as a serious risk. Arguments also exist for concerted cross-border cooperation to control typhoid fever in the southern African region, as it appears that population movements may contribute to disease burden in South Africa.^31^ Ongoing reports of outbreaks in neighboring countries, such as Zimbabwe,^45^ support this as a reason for concern and area of international cooperation. Further data are required to clarify the links between HIV and typhoid fever.^1^ Testing for HIV infection is currently not mandatory and is often not undertaken in patients presenting with *Salmonella* Typhi infection^1^: it has been suggested that HIV infection could be protective against acquiring typhoid fever.^46^ Better elucidation of strain differences within the country at a molecular epidemiological level, to better define imported cases from neighboring states versus those that are locally acquired. Molecular epidemiological analyses will be improved by routine WGS analysis of selected isolates. Given the past history in South Africa of major outbreaks, following the breakdown of systems supplying safe water,^2,3^ high levels of awareness and ongoing active surveillance remain mandatory. Interventions that include targeted vaccination against typhoid fever in high-risk areas may assist in preventing further large-scale outbreaks.

## CONCLUSION

Since the major water projects of the 1980s and 1990s, little has been achieved to further decrease typhoid fever burden in South Africa in the 21st Century. Although the overall burden of typhoid fever has decreased in South Africa over the past few decades, the disease remains present at low endemicity and the country may still experience significant outbreaks. Year-to-year, no further decrease in the incidence rate of culture-confirmed typhoid fever has been noted since 2003. A large migrant population within South Africa, from countries where typhoid fever burdens are high, adds to the local incidence rates. These imported cases, frequently due to MDR *Salmonella* Typhi isolates, which have become endemic to South Africa, complicate disease management. These challenges raise the issue that typhoid fever control is neither simply of national nor subnational concern, but of global relevance. Strategies for vaccination, antimicrobial management and socioeconomic development to combat typhoid fever must, be discussed at this level.

## Supplementary Material

Supplemental table and figures
