# Supplementary figures and images for: The Burden of Typhoid Fever in South Africa: The Potential Impact of Selected Interventions
Source: Am J Trop Med Hyg. 2018 Jul 25;99(3 Suppl):55–63. doi: 10.4269/ajtmh.18-0182 (PMC6128358; doi:10.4269/ajtmh.18-0182)

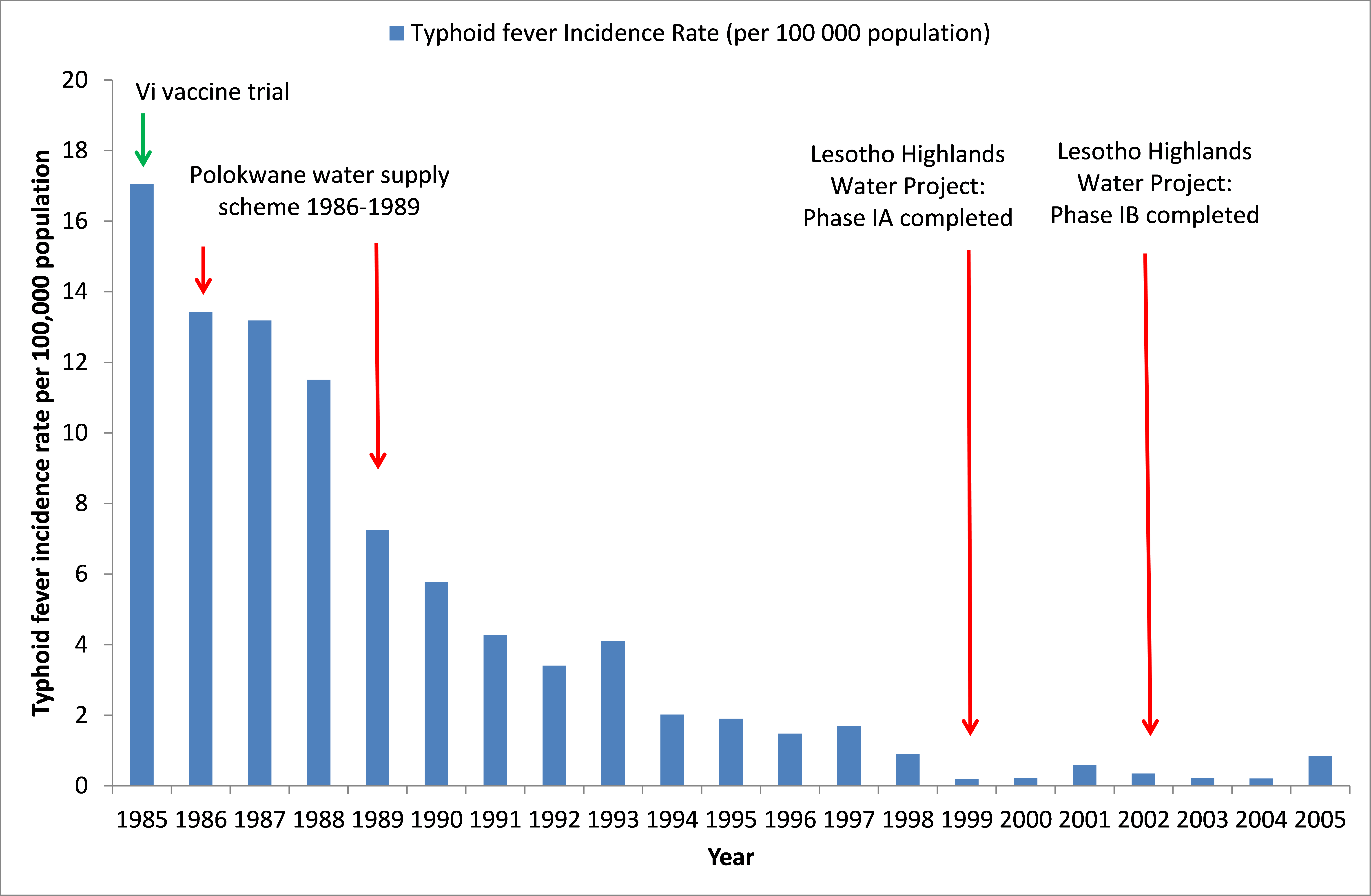

Supplement: Supplementary file 1 [file tpmd180182.SD1.tif]

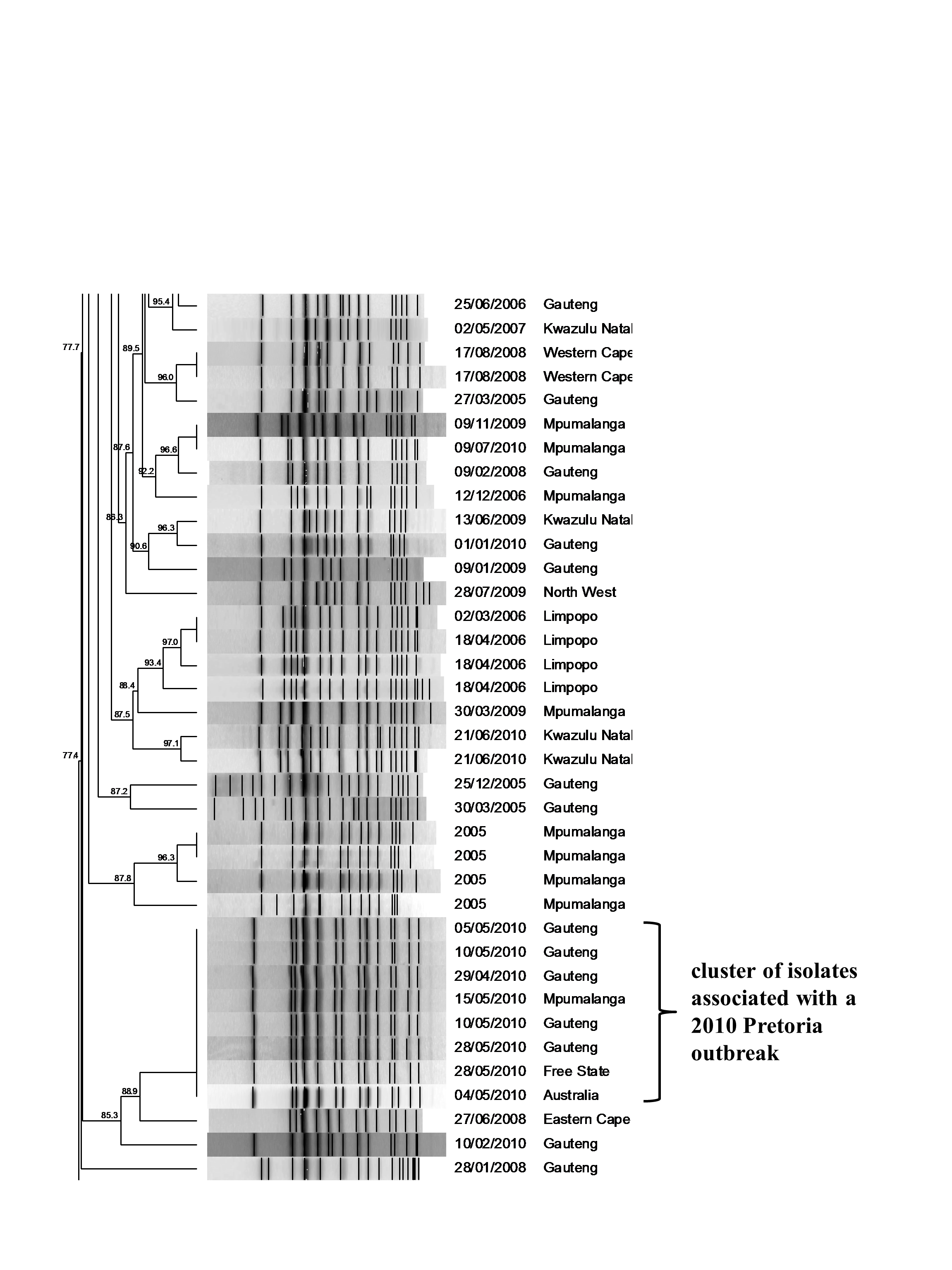

Supplement: Supplementary file 2 [file tpmd180182.SD2.tif]

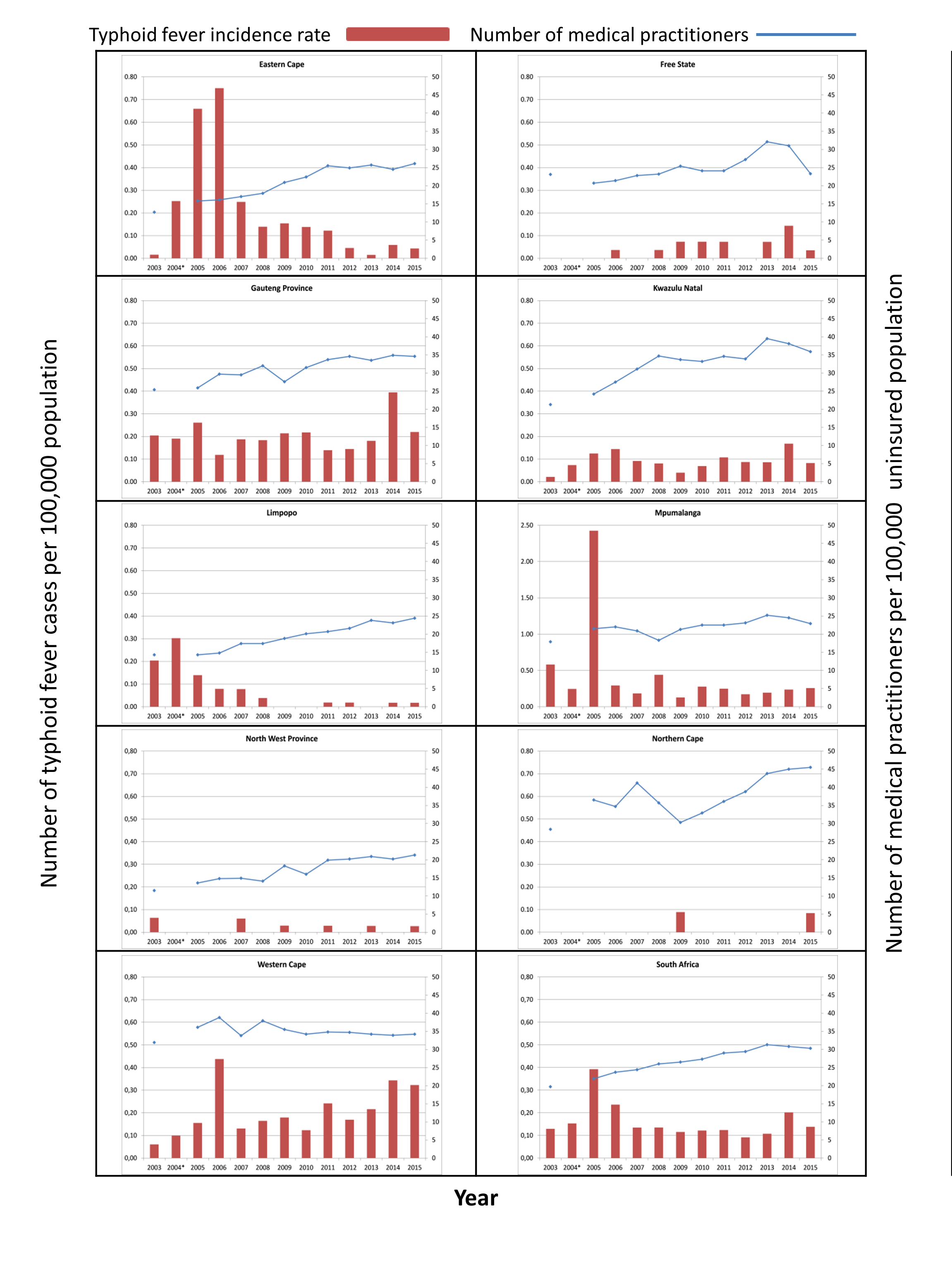

Supplement: Supplementary file 3 [file tpmd180182.SD3.tif]
