# Supplementary material for: The Burden of Typhoid Fever in South Africa: The Potential Impact of Selected Interventions
Source: Am J Trop Med Hyg. 2018 Jul 25;99(3 Suppl):55–63. doi: 10.4269/ajtmh.18-0182 (PMC6128358; doi:10.4269/ajtmh.18-0182)
Supplement: Supplementary file 4 [file tpmd180182.SD4.pdf]

Supplementary table 1. Comparison of access to safe water[19], adequate sanitation[19], laboratory diagnostics expenditure per capita and as a percentage of health care budget by province in South Africa in 2004 (National Health Laboratory Service; unpublished data), compared with culture-confirmed typhoid fever incidence rates in 2004 and 2014.

	Eastern Cape	Free State	Gauteng Province	Kwazulu Natal	Limpopo	Mpumalanga	Northern Cape	North West Province	Western Cape
Percentage of the population with access to safe water by RDP standards[19]	67.4	95.3	95.4	78.4	81.8	80.9	83.6	90.9	96.7
Percentage of the population with access to adequate sanitation by RDP standards[19]	59.9	76.4	88.9	67.6	37.9	57.3	57.5	75.7	91.1
Laboratory Expenditure per Capita, 2004 (South African Rands)	R17	R36	R38	R18	R12	R11	R37	R20	R47
Laboratory Expenditure % Budget, 2004	2.3	3.7	4	2	1.7	1.7	3.8	2.9	4.9
Typhoid fever incidence rate per 100,000, 2004	0.3	0.0	0.2	0.1	0.3	0.2	0.0	0.0	0.1
Typhoid fever incidence rate per 100,000, 2014	0.1	0.1	0.4	0.1	0.0	0.2	0.0	0.0	0.3

*RDP: Reconstruction and Development Programme

Suppl. fig 1. Decreasing incidence rates of typhoid fever from 1985 to 2005, showing timing of interventions including Vi vaccine trial[4] and water projects[4, 26] in high-incidence areas (Limpopo province and Kwazulu-Natal).[12, 13]

Suppl. fig 2. Pulsed-field gel electrophoresis (PFGE) for *Salmonella* Typhi isolates linked an outbreak in Pretoria**.** Snapshot from a dendrogram of PFGE patterns (*Xba*I digestion) for *Salmonella* Typhi, highlighting a cluster of isolates associated with a 2010 Pretoria outbreak.

Suppl. fig 3. The number of culture-confirmed cases per province, compared with the number of public sector medical personnel, per 100,000 population, in South Africa from 2003 - 2015.

The primary axis on the graph for Mpumalanga is not standardized with the others, to allow for incidence rate differences in 2005 when a major outbreak was identified.
